# The Enigmatic Aliphatic Acetogenins and Their Correlations With Lipids During Seed Germination and Leaf Development of Avocado (*Persea americana* Mill.)

**DOI:** 10.3389/fpls.2022.839326

**Published:** 2022-05-03

**Authors:** Álvaro Colin-Oviedo, Sara M. Garza-Aguilar, Luis Martín Marín-Obispo, Dariana Graciela Rodríguez-Sánchez, Víctor Trevino, Carmen Hernández-Brenes, Rocío I. Díaz de la Garza

**Affiliations:** ^1^Tecnologico de Monterrey, Escuela de Ingeniería y Ciencias, Monterrey, Mexico; ^2^Tecnologico de Monterrey, The Institute for Obesity Research, Integrative Biology Unit, Monterrey, Mexico; ^3^Tecnologico de Monterrey, Escuela de Medicina, Monterrey, Mexico

**Keywords:** avocado (*Persea americana* Mill), unusual fatty acids, polar lipids, triacylglycerols (TAGs), glycerolipids, phospholipids, seed germination, leaf development

## Abstract

Lipids in avocados have been widely studied due to their nutritional value and several reported bioactivities. Aliphatic acetogenins are a relevant component of the avocado lipidome and have been tested for several potential food and pharma industries applications. This work followed the evolution of avocado fatty acids (FAs) and aliphatic acetogenins during seed germination and leaf growth. Oil extracts of embryonic axes, cotyledons, and leaves from seedlings and trees were divided to analyze free acetylated acetogenins (AcO-acetogenins), and free FAs. Embryonic axes from germinating seeds contained the highest amount of AcO-acetogenins and FAs; this tissue also accumulated the most diverse FA profile with up to 22 detected moieties. Leaves presented the highest variations in AcO-acetogenin profiles during development, although leaves from seedlings accumulated the simplest FA profile with only 10 different FAs. Remarkably, AcO-acetogenins represented half of the carbons allocated to lipids in grown leaves, while embryonic axes and cotyledons always contained more carbons within FAs during germination. Thus, we hypothesized the use of the AcO-acetogenin acyl chain for energy production toward β-oxidation. Also, α-linolenic and docosahexaenoic acids (DHAs) were proposed as close AcO-acetogenin intermediaries based on a correlation network generated using all these data. Another part of the oil extract was fractionated into different lipid classes before transesterification to profile FAs and acetogenins bound to lipids. Acetogenin backbones were identified for the first time in triglycerides from cotyledons and mainly in polar lipids (which include phospholipids) in all developing avocado tissues analyzed. Seed tissues accumulated preferentially polar lipids during germination, while triglycerides were consumed in cotyledons. Seedling leaves contained minute amounts of triglycerides, and polar lipids increased as they developed. Results from this work suggest acetogenins might be part of the energy and signaling metabolisms, and possibly of membrane structures, underlining the yet to establish role(s) of these unusual lipids in the avocado plant physiology.

## Introduction

Avocado is an ancient angiosperm with origins in tropical and subtropical regions of Mesoamerica. Nowadays, it is consumed worldwide, and Mexico is the principal supplier (Zafar and Sidhu, 2018). The avocado mesocarp accumulates high levels of several vitamins and fatty acids (FAs), and its oil can represent up to 20% of the fresh weight, depending on the cultivar (Takenaga et al., 2008). Therefore, avocado is a rich source of energy and micronutrients (Dreher and Davenport, 2013; Méndez-Zúñiga et al., 2019).

Besides the nutritional benefits of the avocado fruit mesocarp, tissues from the whole fruit contain bioactive compounds, such as lipid derivatives and polyphenols, that could be used for health, cosmetic, and food industry applications (Araújo et al., 2018). Non-edible peel and seed are usually residues; however, their lipidic extracts show potential use for the prevention of infectious diseases as well chronic conditions, such as diabetes and hypertension (Dabas et al., 2013). In addition to poly- and mono-unsaturated FAs (PUFAs and MUFAs), an unusual lipid class in avocado, aliphatic acetogenins, extracted from the seed have shown various bioactive properties (Rodríguez-Sánchez et al., 2013). Aliphatic acetogenins are a group of hydroxylated, odd-chain FA derivatives shown to inhibit microbial growth (Supplementary Table 1; Salinas-Salazar et al., 2017), endospore germination (Rodríguez-Sánchez et al., 2013), *in vitro* cancer cell development (D’Ambrosio et al., 2011), and also have shown antiplatelet and antithrombotic activities in mice (Rodríguez-Sánchez et al., 2015).

According to the majority of unusual lipids, there is scarce knowledge on the metabolism of aliphatic acetogenins and their role in avocado plant development. Nonetheless, previous determination of acetogenin profiles in fruit tissues from 22 different avocado accessions revealed their prevalent presence among the varieties and landraces (Rodríguez-López et al., 2015). Acetogenins are also the most abundant lipid group in the seed lipidome, and, in mesocarp, they are mostly contained inside idioblasts, oil-containing specialized cells (Rodríguez-López et al., 2017).

Avocado seeds are not considered oily; as they develop, they accumulate non-structural carbohydrates, mostly starch, which constitutes up to 33% of the fresh weight (Weatherby and Sorber, 1931; Bora et al., 2001). By contrast, during fruit development, seeds accumulate oil to the extent of up to 0.35% of their fresh weight during the first 110 days after bloom (Ge et al., 2021). Linoleic, oleic, and palmitic acids are the principal FAs accumulated in avocado seeds (Ge et al., 2018). Previously, we have determined that a third of the diacylglycerols (DAGs) and triacylglycerols (TAGs) in the seed lipidome contain FAs with odd-carbon chains. This indicates a possible pool of precursors to maintain acetogenin synthesis (Rodríguez-López et al., 2017). Accordingly, acetogenin accumulation occurs during seed development (Rodríguez-López et al., 2017).

Recently, we characterized the acetogenin profile in avocado leaves, which can produce the most characterized acetogenins, but conversely to seeds, they accumulate mostly AcO-persin (Rodríguez-Sánchez et al., 2019). Similar to acetogenins from seeds, various biological activities have been associated with avocado leaves extracts, including anticonvulsant, antihypertensive, and antioxidant properties (Ojewole and Amabeoku, 2006; Irawati, 2015; Polat Kose et al., 2020). However, avocado leaf consumption has also been associated with toxicity in mammals, attributed to its high amounts of AcO-persin (Butt et al., 2006).

Mesocarp has been the principal target for lipid studies in avocado plants, and knowledge about leaf lipids is limited. The TAG composition in leaves has been determined in diverse plants like *Arabidopsis*, *Brachypodium* (Yang and Ohlrogge, 2009), and *Malus x* Snowdrift (Lin and Oliver, 2008). C18 FAs are predominant in Arabidopsis leaves, while C16, C18, and C18:1 were the principals in *M.* snowdrift. Unlike seeds, leaves do not accumulate TAGs as energy storage; TAGs produced in leaves appear to function as a transient pool of FAs during membrane turnover and modification (Chapman and Ohlrogge, 2012).

To gain knowledge about acetogenin production in plant development, the present study aims to describe the parallel progression of acetylated acetogenins (AcO-acetogenins) and FA contents during the seed germination in avocado, following embryo’s cotyledons and axis and seedling leaves. Tree leaves at different developmental stages were also included to complement the study. Additionally, we performed a partition of these lipid extracts to separate TAGs, MAGs, DAGs, and polar lipids (PL) to elucidate the presence and profile of FAs and acetogenins within complex lipids. Together, all these data show for the first time the most exhaustive characterization of lipid molecules in avocado during germination and leaf development.

## Materials and Methods

### Plant Material

Avocado (*Persea americana var. Hass*) seeds were obtained from fresh ripe fruits from a local shopping center (H-E-B Mexico). As soon as seeds were collected, they were washed with Terg-a-zyme (ALCONOX^®^, United States), left to air-dry, and stored at 4°C. Seed coats were removed and placed in plastic containers with distilled water (approximately 2 cm deep). Imbibition took place at 25°C in a storage room, under a 16:8 light and dark cycle. Seeds were collected and dissected, with separation of the embryonic axis (EA) and cotyledon, on 0, 7, 14, 21, and 28 days after imbibition (dai). Seedlings were obtained by extended imbibition under the same conditions mentioned. Once leaves emerged (approximately after 2 months of imbibition), they were sampled. A total of three different leaf sizes were collected, varying from 1 to 5 cm. They were classified as young leaf (YL, 66 days), middle-grown leaf (MGL, 72 days), and full-grown leaf (FGL, 78 days). A set of two *P. americana* var. Hass 4-year-old trees were used for leaf sampling. The trees were kept outdoors in a greenhouse at Monterrey, Mexico, and watered every second or third day. Three different leaf sizes were collected, varying from 5 to 10 cm. These leaves were also classified as YL, MGL, and FGL based on their size. An example of the plant material used is shown in Supplementary Figure 1.

### Lipid Extraction

A modification of Folch’s method (Iverson et al., 2001) was implemented to obtain a raw lipid extract that allowed further enrichment for Acetogenins or FA. Briefly, 1 ml of a 1:1 mixture of dichloromethane:methanol was added to plant material (10–100 mg) and ground using a FastPrep homogenizer (MP Biomedicals, United States). Phases were separated by centrifugation (5 min, 8,000 g), the organic phase was stored, and this extraction process was repeated two times using 0.3 ml of fresh solvent. The final extract (1.5 ml) was divided into three aliquots for two studies: two aliquots for acetogenin enrichment and FA enrichment (*Study I*), and one aliquot for lipid fractionation (*Study II*). The aliquots were evaporated at 55^°^C using vacuum-assisted centrifugation (CentriVap, LABCONCO, United States) and stored at –20°C.

### AcO-Acetogenin Enrichment *(Study I)*

Previously developed methods for acetogenin enrichment were slightly modified (Rodríguez-López et al., 2015). Extracts were dissolved in 1 ml of a Hexane:Water mixture. Next, the mixture was vortexed, and the two immiscible phases were separated by centrifugation (5 min, 8,000 g). Finally, the organic phase was collected, and the same procedure was repeated two more times with 0.5 ml of fresh hexane to the remaining aqueous phase. Supernatants were pooled and evaporated at 55^°^C. The remaining extract was reconstituted in isopropanol and filtered through a 0.22 μm membrane.

### Determination of AcO-Acetogenins by HPLC-DAD

Chromatographic methods for acetogenin determination were based on previous work (Rodríguez-López et al., 2015). A Waters UHPLC (Acquity System, Waters Corporation, United States) system coupled to a DAD detector was used. Detection was carried out at wavelengths of 220 and 208 nm. HPLC grade water (Solvent A) and methanol (MeOH, Solvent B) composed the mobile phases. Pumps were set to 0.8 ml/min, with a gradient of 20/80% (A/B) for 15 min, 5/95% 10 min, 0/100% 5 min, and lastly, equilibration at 20/80% for 20 min. Separation was carried out using a Phenomenex (United States), Synergi 4μ Hydro-RP 4 μm 4.6 × 250 mm.

### Lipid Classes Fractionation (*Study II*)

The third subsample was subsequently fractionated according to a previously described method (Agren et al., 1992). Using a solid-phase extraction aminopropyl column (500 mg, 3 ml, Bond Elut NH2, Agilent Technologies Inc., CA, United States). Cholesteryl nonadecanoate (400 ppm, Nu Chek Prep Inc., MA, United States) and 1,2-Diheptadecanoyl-sn-glycerol-3-phosphorylcholine (400 ppm, Abcam, Cambridge, United Kingdom) were added as internal standards. Five different lipid classes with different polarities were collected and identified according to the elution of standards as phytosterol esters (F1), triglycerides (TAGs, F2), mono- and di-glycerides (MAG + DAG, F3), free FAs (F4), and polar lipids, which include phospholipids and glycerolipids (PLs, F5). After elution, the resulting fractions were dried in a Centrivap vacuum concentrator (Labconco, MO, United States) and reconstituted with a toluene-hexane mixture (0.5 ml, 1:1 v/v).

A standardized avocado seed extract Avosafe^®^, 94.74% free AcO-acetogenins (Rodríguez-Sánchez et al., 2019) was fractionated through the previously described scheme to determine in which fraction the native free acetogenins eluted (Supplementary Table 1). Further derivatization (described below) of each fraction allowed us to hypothesize which lipid class, and the resulting fatty acid methyl ethers (FAMEs), or acetogenin methyl ethers (AcOMEs) were potentially bound (Supplementary Figure 2). Since 89% of Avosafe eluted in the sterols, MAG + DAG, and free FAs, we only collected the TAG and polar lipids (PL) fractions for further acetogenin analyses of our samples. For FAS, the MAG + DAG fraction was also analyzed to characterize the glycerolipids in avocado.

### Fatty Acids and Acetogenin Derivatization

Avocado subsamples (from *study I* or *II*), AcO-avocadene, which is an NMR-characterized free acetogenin analytical standard (Rodríguez-Sánchez et al., 2013), and Avosafe^®^ were independently derivatized (Castillo et al., 2020). Undecanoic acid (200 ppm, Nu Chek Prep Inc., MA, United States) was used as an internal quantification standard.

### Analysis of External Standards by GC-MS

A standard mixture containing 39 FAMEs (GLC 566, Nu Chek Prep Inc., MA, United States) or standardized AcOMEs obtained in the previous section (*Study II*) were analyzed by GC–MS chromatography (Agilent 7890B/5977A, Agilent Technologies Inc., CA, United States) to identify the lipids. A previously developed method was used (Castillo et al., 2020). Having as reference fragmentation patterns of FAMEs (Jabbar et al., 2014; Busta et al., 2016) and Mclafferty rearrangement characteristic of esters (McLafferty, 1956; Weiler, 1972), the expected fragmentation patterns of each of the free AcO-acetogenins reported here (based on their structure) were matched to their observed mass spectra (Supplementary Figure 2).

### Sample Analysis by GC-FID

Fatty acid methyl ethers and AcOMEs profiles present in oil extracts from avocado (from *study I* or *II*) were determined by gas chromatography using a flame ionization detector (GC- FID 6850A, Agilent Technologies Inc., CA, United States). The column and chromatographic conditions were those used for GC-MS (Castillo et al., 2020). Quantification by GC-FID was determined by comparing response factors of analytical standards, according to the AOAC method 996.06 (AOAC, 2000).

### Data Analysis

Data from AcO-acetogenin and FA determinations were expressed as means ± standard error (SE) unless otherwise specified. Statistical differences among treatments were determined using ANOVA. Additionally, the Fisher *post hoc* test was used for grouping. These statistical analyses were carried out on Minitab^®^ version 19.2020.1 software.

For principal components (PCA), correlation, and network analyses, molecular data were first normalized by the molecular weight and to the sum of the total mass figure detected. That is, (x_*i*_/mw_*i*_) × 1,000/sum(x). The normalization was performed for acetogenins and FAs separately. For PCA, data were first logarithm base 10 transformed. The calculations were performed in R.^1^ For the hierarchical clustering in the correlation analysis, *Euclidean* distance and *Ward* agglomeration was used. The package *network* in R was used to generate the metabolite network.

## Results

During avocado seed imbibition, Phase I of germination, where water is absorbed, occurred between 1 and 15 dai (Supplementary Figure 3). Afterward, the water content was relatively constant, and at 21 dai, 50% of the seeds presented EA emergency before the radicle protrusion, reaching germination. Thus, the last sample in this study (collected at 28 dai) is considered in the post-germination stage. Finally, the second step of water absorption was observed as a signal of seedling establishment, where only the developing EAs increased in humidity.

### The Embryonic Axis Accumulates a Full AcO-Acetogenin Profile During Avocado Seed Germination

**FIGURE 1:**
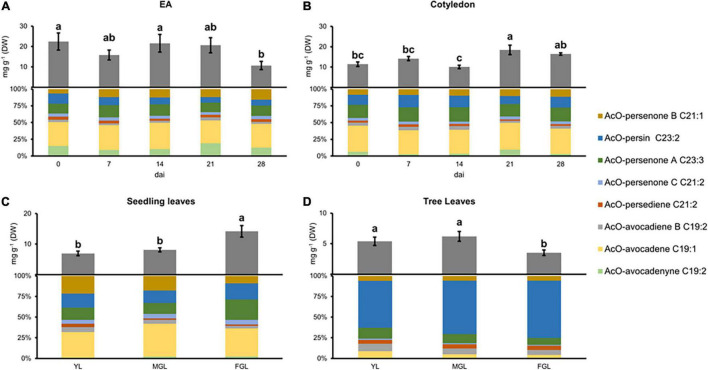
Acetogenin content and composition in different avocado tissues. **(A)** EA, Embryonic axis. **(B)** COT, Cotyledon. **(C)** SDL, Seedling leaves. **(D)** TRL, Tree leaves. Error bars represent standard error (SE), different letters indicate statistical differences between treatments (ANOVA, Fisher LSD, *P* < 0.05). *n* = 4 for EA and Cotyledon; *n* = 3 for leaves. EA embryonic axis.

Acetylated-acetogenins had been profiled previously in developing avocado cotyledons; however, this is the first time they are profiled in EA. AcO-acetogenin levels during the first 21 dai were consistently higher in EA when compared to cotyledons (*P* < *0.05*, paired *t*-test, Figures 1A,B). EA lost 46% of total AcO-acetogenin content at 28 dai compared with 0 dai; conversely, cotyledons had small fluctuations during imbibition with similar concentrations measured at 0 and 28 dai. Regarding individual AcO-acetogenins, AcO-avocadene was the most abundant component in both tissues comprising up to 40% of the total pool (Supplementary File 1). Other components of the pool were distributed similarly between the two tissues, except for AcO-persenone C in cotyledon, where it represented up to 20% of the extract, while in the EA, it comprised less than 5%, while AcO-avocadenyne had more contribution to the EA pool.

### Avocado Leaf Development Leads to AcO-Persin Accumulation

Acetogenin profiles in seedling and tree leaves had a dynamic accumulation during their growth. The overall AcO-acetogenin content in seedling leaves significantly increased as leaves grew, doubling its initial concentration (Figure 1C). On the other hand, AcO-acetogenin contents in tree leaves did not change during the first two growing sizes but significantly decreased in full-grown leaves (FGL) (Figure 1D).

AcO-avocadene was the most abundant component of seedling acetogenin extracts representing up to 40% of the total pool. The second-largest component was AcO-persenone B (20%); however, it suffered a 10% reduction during the last stage, while AcO-persenone A had a simultaneous increment. Leaves from trees showed an AcO-acetogenin content reduction during the last stage except for AcO-persin, which contrary to leaves from seedlings, was the most prevalent acetogenin, representing up to 70% of the acetogenin pool in FGL. AcO-avocadenyne was not detected in young leaves (YL) from seedlings, and, in addition to AcO-persenone C, it was also absent in tree leaves.

### The Embryonic Axis Is Composed by a Diverse and Complex Fatty Acid Profile

**FIGURE 2:**
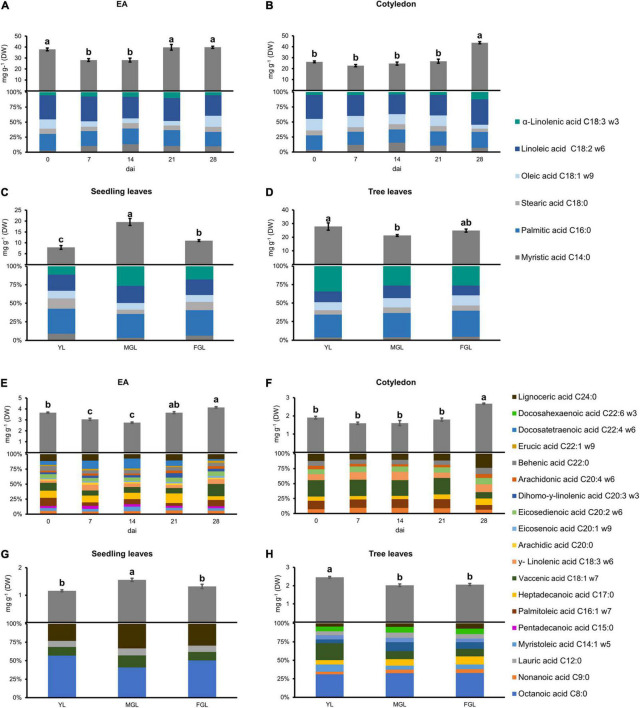
Major and minor FA content and composition in different avocado tissues. **(A,E)** EA. **(B,F)** COT. **(C,G)** SDL leaves. **(D,H)** TRL. Error bars represent SE, Different letters indicate statistical differences between treatments (ANOVA, Fisher LSD, *P* < 0.05). *n* = 4 for EA and Cotyledon; *n* = 3 for leaves. EA embryonic axis.

Fatty acids were profiled in germinating cotyledons and EAs to correlate them to the acetogenin accumulation. The FA pool was divided into major and minor FAs based on their abundance, <1 mg/g DW for minor FAs (Figure 2). The accumulation of both major and minor FAs changed little in both seed tissues. EA had a general decrease in FAs at 7 and 14 dai, and it recovered the initial concentration later in germination (Figures 2A,E). Interestingly, cotyledons had an increase of 180% on the last day of sampling when compared to previous stages (Figures 2B,F). Such increase was due to PUFAs accumulation, such as linoleic and α-linolenic acid. Minor FAs accumulated within the EA pool harbored more very-long-chain FAs (20, 22 carbon chains) and their unsaturated kinds (Figure 2E). EA content showed a 25% decrease in minor FAs at 7 and 14 dai, but later it regained its initial amount. Moreover, major FA content in cotyledons showed a stable concentration until the 28 dai, where it presented a 1.8-fold increase (from 26.81 to 42.76 mg/g DW). Individual FA species followed the same concentration pattern as the total amounts. Linoleic acid was the most abundant major FA species, representing up to 40% of the extracts in both tissues.

The diversity of minor FAs was considerably greater in the EA than in cotyledon (15 vs. eight different species). The total pool of minor FAs followed the same pattern as their major counterpart in both tissues (EA and cotyledon). EA had very-long-chain FAs (20, 22, and 24 carbon chains) with up to four unsaturated carbons (Figure 2E). We also observed the accumulation of two odd-chain FAs, penta- and heptadecanoic acids. EA profile showed no principal species until 28 dai, where vaccenic acid comprised 21% of the pool. Vaccenic acid was also the most abundant species in the cotyledon minor FA pool (30%) until 28 dai when it was significantly reduced.

### Fatty Acid Reduction in Avocado Leaves Concurs With Acetogenin Accumulation

Young leaves in seedlings accumulate lower FAs levels than those from the tree; they also had a simple profile in comparison (Figures 2C,G). Leaves from seedlings only accumulated four minor FAs, mainly octanoic acid followed, intriguingly, by very long-chain FAs (Figure 2G). Total FAs in seedling leaves had a 4-fold increase in major FA levels during the second stage, which later reverted. On the other hand, YL from trees accumulated up to 30.3 mg/g DW of major FAs, and we observed a 20% decrease of the total FA pool during the second and third developing stages. Tree leaves presented a broader FAs profile than seedlings (10 vs. four species) (Figure 2H). Seedling leaves contained mostly medium and long-chain FAs, and their level of unsaturation was lower than tree leaves, which always had more than 50% of the total FAs pool unsaturated; in fact, the level of docosahexaenoic acid (DHA) (C22:6) significantly increased by 1.6 as the tree leaf developed. Interestingly, a short-saturated FA, octanoic acid, was the most prevalent among the minor FAs in all leaves measured.

### Dynamics of Lipid Elongation and Unsaturation During Seed Germination and Leaf Development

**FIGURE 3:**
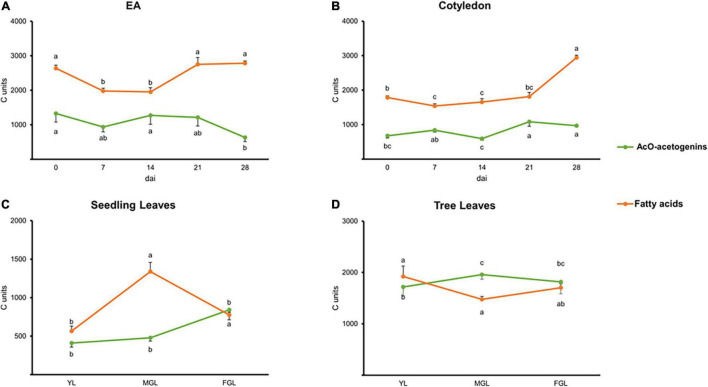
Carbon units are allocated into acetogenins and FAs. **(A)** EA. **(B)** Cotyledon. **(C)** Seedling leaves. **(D)** Tree leaves. Points indicate mean values. Error bars indicate SE. Error bars and statistical groups are shown above the line for FA and below the line for acetogenins. Different letters indicate significant statistical differences between treatments (ANOVA, Fisher LSD, *P* < 0.05). *n* = 4 for EA and Cotyledon; *n* = 3 for leaves. EA embryonic axis.

**FIGURE 4:**
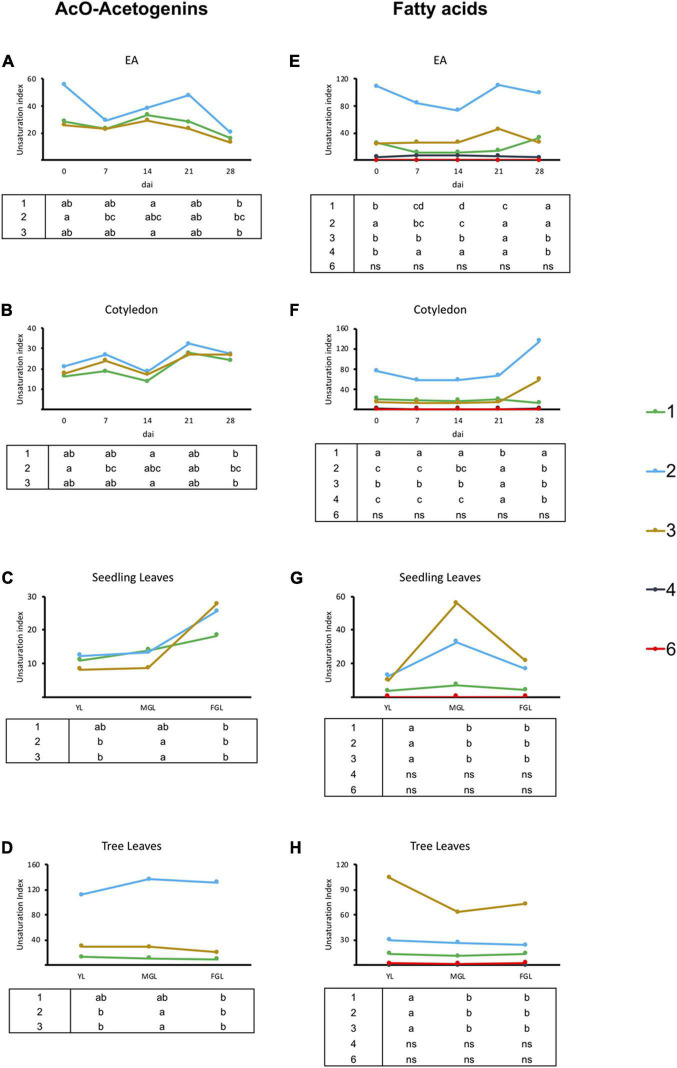
Unsaturation index for acetogenins and FAs in avocado seeds and leaves. **(A–D)** Unsaturation indices for acetogenin content of EA, COT, SDL, and TRL, respectively. **(E–H)** Unsaturation indices for FA content of EA, Cotyledon, Seedling leaves, and Tree leaves respectively. Each point represents mean values. The tables below graphics indicate statistical differences among groups for each unsaturation degree. Different letters indicate significant statistical differences between treatments (ANOVA, Fisher LSD, *P* < 0.05). *N* = 4 for EA and Cotyledon; *n* = 3 for leaves. EA embryonic axis.

To obtain general knowledge of the avocado lipids metabolism during the two processes explored in this work, we calculated the number of carbons allocated to the lipids and their unsaturation level distribution, regardless of specific molecules. A “carbon content” was considered by taking into account the number of carbons within the lipid chain multiplied by the number of micromoles of the lipids detected per gram of dry tissue (μm/g DW). Additionally, a saturation degree was calculated by pooling the μm/g DW of lipids by their number of unsaturated carbons within the aliphatic chain of all lipids in an unsaturated class (major FAs or acetogenins, Figures 3, 4).

During seed germination and leaf development, the number of carbons directed toward acetogenins or major FAs seemed to inversely correlate as observed with individual lipidic species (above). Cotyledons appeared to maintain acetogenin and FA synthesis even after root protrusion (Figure 3B). EA and cotyledon’s total carbon content (65–85% of carbons) was mainly harbored by major FAs. On the contrary, fully photosynthetic leaves from trees had an even distribution of their carbons in these two lipids throughout development. Meanwhile, developing leaves from seedlings had a peak of carbons going to major FAs in MGL, followed by a steep decrease with a concomitant increase in carbons within acetogenins (Figures 3C,D).

Unsaturation degree in lipids can be crucial for their function; acetogenins analyzed here presented from up to three unsaturations in their acyl chain, including two acetogenins with a terminal triene (Supplementary Table 1). Except for EA, all tissues had a constant unsaturation distribution through time (Figure 4). Monounsaturated acetogenins were prevalent, while leaves sampled from trees contained significantly more two-unsaturated acetogenins, mainly due to the high prevalence of AcO-persin (C23:2) (Figure 4D). Regarding major FAs, the only change in the unsaturation profile occurred during the last week of seed sampling, with an increase in monounsaturated FAs with a concomitant decrease in unsaturated carbons in both cotyledons and EAs. Interestingly, seeds contained a prevalent pool of saturated FAs (53–77%). Conversely, young developing leaves in seedlings had a smaller saturated pool, which slightly increased as they developed (25–39%), while tree leaves had half of the major FA pool saturated (Figures 4C,G).

### Docosahexaenoic Acid Is a Potential Precursor in Acetogenin Biosynthesis Along With α-Linolenic and Linoleic Acids

**FIGURE 5:**
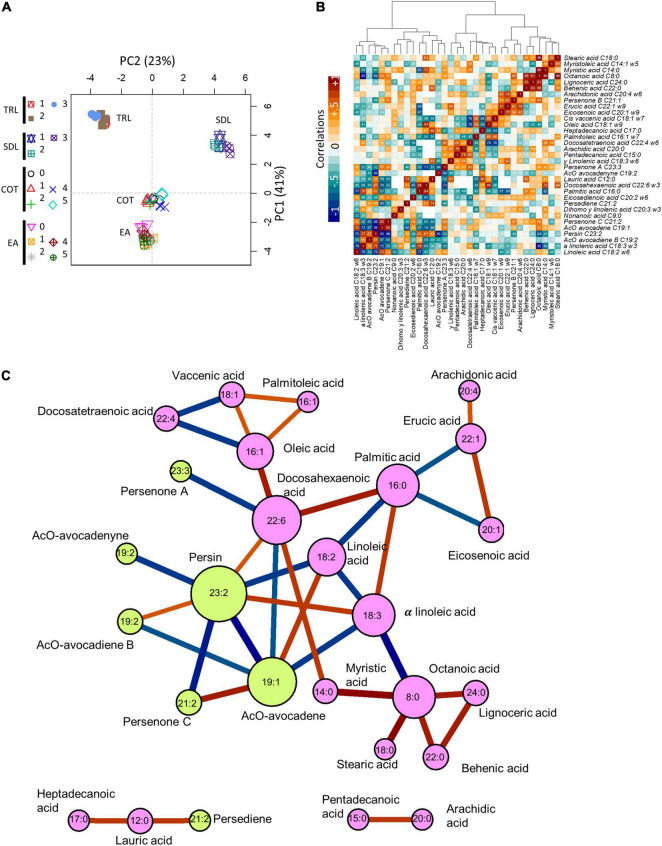
Explained experimental variance and correlation among metabolites. **(A)** First two principal components associated with main factors, EA, COT, SDL, and TRL in the first component (vertical), and TRL and SDL in the second component (horizontal) after logarithm transformation. **(B)** Correlation matrix among metabolites clustered by absolute correlation, Euclidean distance, and ward agglomeration. Numbers represent absolute correlation; colors represent correlation value as indicated. **(C)** The network formed after removing absolute correlations lower than 0.66. Edges color (red, positive; blue, negative) and width represent the correlation. Nodes represent metabolites as labeled; size is proportional to connections. Pink for fatty acids and green for acetogenins. Data were obtained from 56 samples and 112 determinations.

We used all the individual data generated in the first part of this study to perform multivariate and correlation analyses (Figure 5). The first two components shown in the PCA explained 64% of the variance (Figure 5A). PC1 clearly separated EA, cotyledon, and leaf tissues, while the resolution between seedling and tree leaves was evident in PC2. Absolute correlations of all measured lipids are depicted in Figure 5B. Several significant negative correlations can be observed. Avocadenyne, Avocadene, Persenone C, Persenone B, and Persenone A showed the most negative correlation with FA species, indicating close metabolic relationships. These AcO-acetogenins correlated highly negatively with three groups of FAs: Short-chain FAs (such as myristic acid), polyunsaturated FAs (such as α-linolenic acid), and long-chain FAs (such as lignoceric acid).

A subset composed of the highest correlations (|*r*| > 0.66) was used to build a network that allowed us to graphically depict the best relationships found among these lipids. The network shows the clustering of unsaturated FAs, monounsaturated and polyunsaturated FAs (Figure 5C). Also, acetogenins are grouped into a cluster of their own. Interestingly, linoleic, α-linolenic, and DHAs were the FA species closer and negatively correlated to the acetogenin cluster. Both AcO-persin (C23:2) and AcO-avocadene (C19:2) were the most correlated acetogenins within the network. Finally, we can observe one single, separated cluster containing one acetogenin (AcO-persediene) directly linked to lauric acid with a positive correlation that did not directly relate to the other compounds with the threshold used. Networks representing the correlations from each individual tissue contributing to the overall network are depicted in Supplementary Figure 4 where specific differences can be observed (e.g., more connections in tree leaves and less connections in EA).

### Polar Lipids Containing Fatty Acids Dramatically Increased During Development in Avocado Cotyledons and Seedling Leaves

**FIGURE 6:**
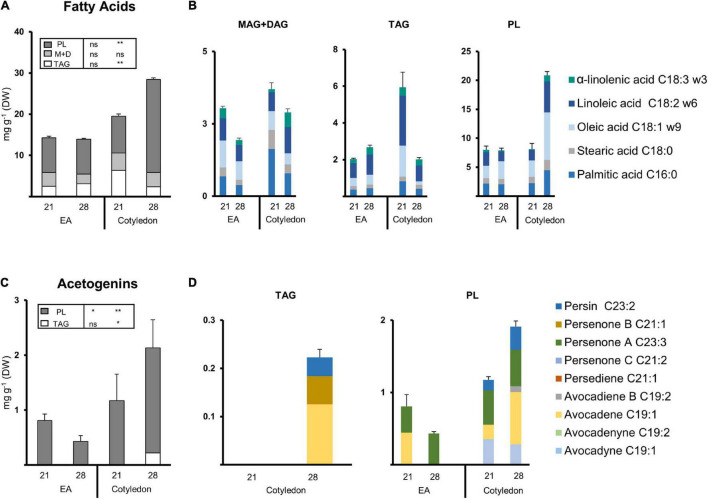
Lipid composition of the EA and cotyledon extracts from two developmental stages. **(A)** Composition of the fatty acid extracts from EA and COT at 21 and 28 dai. **(B)** Fatty acid composition of lipid fractions from the EA and Cotyledon at 21 and 28 dai (Left, TAGs; Middle, MAG + DAGs Right, PLs). **(C)** Composition of the Acetogenin extracts from EA and COT at 21 and 28 dai. **(D)** Acetogenin composition of lipid fractions from EA and COT at 21 and 28 dai (Left, TAGs; Right, PLs; no FAMEs were detected in the TAG fraction for cotyledon at 21 dai). Bars represent an average of 3 samples. Tables contain statistical differences in between treatments (ANOVA, Fisher LSD, *P < 0.05, **P < 0.01; ns = non-significant). Error bars indicate Standard Error. PL, polar lipids; MAG, monoacylglycerol; DAG, diacylglycerol; TAG, triacylglycerol. *N* = 3.

**FIGURE 7:**
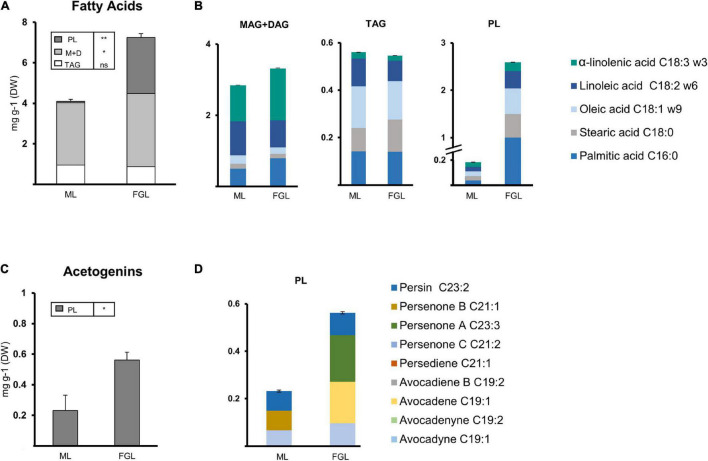
Lipid composition of the leaf extracts from seedlings. **(A)** Composition of the Fatty acid extracts from ML and FGL. **(B)** Fatty acid composition of lipid fractions (Left, TAGs; Middle, MAG + DAGs; Right, PLs). **(C)** Composition of the Acetogenin extracts from ML and FGL. **(D)** Acetogenin composition of lipid fractions. Bars represent an average of 3 samples. (ANOVA, Fisher LSD, *P < 0.05, **P < 0.01; ns = non-significant). Error bars indicate Standard Error (SE). PL, polar lipids; MAG, monoacylglycerol; DAG, diacylglycerol; TAG, triacylglycerol. *N* = 3.

Extracts from selected seed and leaf tissues were fractionated (*Study II*). TAGs, MAG + DAG, and PL fractions were collected and characterized (Figures 6, 7) to explore the potential presence and dynamics of FAs and acetogenins within these lipids during seed and leaf development. Further derivatization of the different extracts allowed us to point to the lipid class in which FAMEs or acetogenin methyl esters/ethers (AcOMEs) were potentially bound (see “Methods”).

Seed tissues of 21 and 28 dai and seedling leaves (ML and FGL) were selected for these analyses. PL was the fraction harboring more FAs in cotyledon and EA, comprising at least 50% of the FAs measured (Figure 6A). Cotyledons showed a significant increase (two-fold) of FAs in PL fraction from 21 to 28 dai. As the FA content within PL from cotyledons increased over time in EA, FAs in TAGs were significantly reduced. The FA content in the MAG + DAG fraction remained constant in both cotyledon and EA. Looking at the distribution of FAs species, during the last stages of germination, the different fractions of the EA retained a similar composition of Major FA (Figure 6A). Conversely, TAGs decreased 1.86-fold in cotyledons. The most abundant species in both tissues were palmitic, linoleic, and oleic acids.

Developing seedling leaves showed a different distribution of lipids than germinating seeds. FAs were mainly distributed in the MAG + DAG fraction with a steep increase in PLs as the leaf grew and a constant minuscule TAG pool. Both MAG + DAG fractions of the MGL and the FL shared the same Major FA content and profile (Figure 7A). The TAG fraction in MGL and FGL was also conserved between the two stages, with a similar composition and a content. Twenty nine-fold that of MAG + DAG fractions. The PL fraction showed minute amounts of FAs during the MG stage (<0.1 mg/g DW). Then, a drastic increase was observed in the FGL stage, where the FA content in PL rose to 2.6 mg/g DW. Palmitic acid was the most abundant component of the PL fraction of FGL (40%), while the other Major FAs were equally distributed in the remaining part of the fraction.

### Avocado Triacylglycerols and Polar Lipids Possibly Hold Acetogenin Moieties

Acetogenin methyl esters/ethers were only analyzed in TAGs and PLs since the majority of free AcO-acetogenins eluted in MAG + DAG fraction. Even though only a small percentage of Avosafe^®^ [concentrated free AcO-acetogenins] eluted along with TAG and PL fractions (Supplementary Table 2), we calculated a threshold concentration based on this percentage applied to the number of free acetogenins quantified in the paired extract from Study I (Figure 1). All results presented for acetogenins in this section were above the threshold from 2.26- to 6.64-fold, showing that the molecules detected were mostly part of the TAGs and PLs.

The PL fraction contained acetogenin backbones in all selected samples, and remarkably, we could distinguish some bound to TAG in cotyledons at 28 dai (Figure 6C). Avocadene was more than half of the pool, followed by persenone B and persin in the small number of acetogenins detected in TAGs (Figure 6D). On the other hand, in seeds, PL fractions suffered a significant reduction in EA while showing a concurrent increase in cotyledon (Figure 6C). During 21 dai PLs from EA contained mainly persin and avocadene in equal amounts, and during 28 dai, persin was the only acetogenin detected. The PL fraction of cotyledon had a significant increase during 28 dai, and its profile indicates the increase was due to a 3.6-fold increment in avocadene. Interestingly, acetogenin backbones in this fraction were not from any of the C21 acetogenins and only a minimal amount of an intermediate unsaturation degree was detected in the cotyledon of 28 dai (Figure 6D).

Acetogenins were detected only in the PL fraction of leaves, showing an increase of 2.4-fold during development (Figure 7C). Avocadyne and persin remained stable in both stages; however, persenone B (21:1) disappeared in FGL while avocadene (19:1) and persenone A (23:1) appeared in this stage constituting 35.18% of the pool.

## Discussion

### Acetogenins Seem to Be Ubiquitous in the Avocado Plant and Are Components of Storage and Polar Lipids

Lauraceous acetogenins were first reported more than 40 years ago in avocado leaves (Chang et al., 1975), and these odd-chain lipids have been detected in whole seed, mesocarp, peel (Rodríguez-López et al., 2015, 2017), and grown leaves (Rodríguez-Sánchez et al., 2019). In this study, we also report the continuous presence of acetogenins in germinating embryos and developing leaves. Hence, one wonders about the possible roles in avocado physiology. Previous studies have classified persin as a defense molecule to contend with herbivorous leaf consumption and fungal pathogens (Kobiler et al., 1993; Ardi et al., 1998; Butt et al., 2006). Moreover, chitosan treatment on avocado fruits increased the antimicrobial activity of their extracts against *Choletotrichum gloesporoiodes*, and it also elicited the upregulation of two genes correlated with persin accumulation (Xoca-Orozco et al., 2019). The reported acetogenin antimicrobial activities against *Listeria monocytogenes* (Salinas-Salazar et al., 2017) and *Clostridium sporogenes* (Rodríguez-Sánchez et al., 2013) also support their possible role as defense molecules against biotic stress. However, the magnitude of acetogenin accumulation in EA and cotyledons (Rodríguez-López et al., 2015; Rodríguez-Sánchez et al., 2019), and its continuous presence in all tissues during development, suggests that defense might not be their only function.

Even though aliphatic acetogenins have only been reported in the avocado plant, unusual, hydroxylated lipids, are not unique to the *Persea* genus. Hydroxylated and epoxygenated FAs were characterized in *Stokesia laevis* (Hatanaka et al., 2004), *Euphorbia lagascae* (Bafor et al., 1993), *Ricinus communis* L. (Burgal et al., 2008), *Glycine max*, *Vernonia galamensis*, and *Arabidopsis* seeds, and they were generally associated with pathogen defense (Li et al., 2010).

It was also observed that during initial *Vernonia* seed development, unusual FAs contents were minimal, while mature seeds accumulated up to 70% of the total seed FAs. Acetogenin accumulation during avocado seed development shows a similar tendency (Rodríguez-López et al., 2017). Besides their possible roles in plant defense (Li et al., 2010), the plant could use unusual FAs as an early form of energy, especially during germination. Significant increases of hydroxy-FAs have been detected during the germination of cucumber, soybean, tobacco, and rape; moreover, free hydroxy-FAs were more common than other free FAs in cucumber cotyledons, suggesting their preferential release from lipids to feed β-oxidation (Feussner et al., 1995). During germination, AcO-acetogenins from EA maintained a two-fold content in comparison to cotyledons until the end of germination (28 dai), when significantly decreased while FAs pool increased. Nothing is known about AcO-acetogenins’ metabolic fate and degradation.

Acetylated-acetogenins quantified in the first part of this work were extracted using solvents without any treatment for their release from other molecules; thus, they are believed to be free within avocado cells. Nonetheless, they contain a long unsaturated acyl chain; therefore, they could also be part of lipid molecules. To test this hypothesis, some of the lipid extracts were fractionated, and TAGs and PLs were analyzed for acetogenin presence; after fractionation, the acyl chain was released by transesterification. Thus, acetogenins depicted in Figures 6, 7 constitute the previously bound form of these molecules, apart from the free form (with an AcO head).

Acetogenins presence was detected in TAGs only in the tissue with the most acetogenin accumulation. While barely detected by our method, this finding is important for considering the possible roles of these lipids in the physiology of the plant. TAGs are storage molecules that can be broken down to supply carbon and energy to a plethora of anabolic routes or used for PL biosynthesis; we were able to detect three acetogenins in TAGs from cotyledons one of each known acyl chain (17, 19, and 21 carbons). The presence of this odd acyl chain in TAGs further supports the idea of their use for more roles than plant defense. Moreover, acetogenins were also consistently found in PL fractions from all tissues, phospholipids elute in this fraction. Besides their structural role in cell membranes, phospholipids are active lipids implicated in different signaling processes of plant physiology, such as plant defense and environmental response (Laxalt and Munnik, 2002; Ruelland et al., 2015). Even though further studies are needed to evidence this possible role, the identification of acetogenins in the PL fraction hints at their involvement in unknown signaling mechanisms and also as part of avocado cell membranes.

**FIGURE 8:**
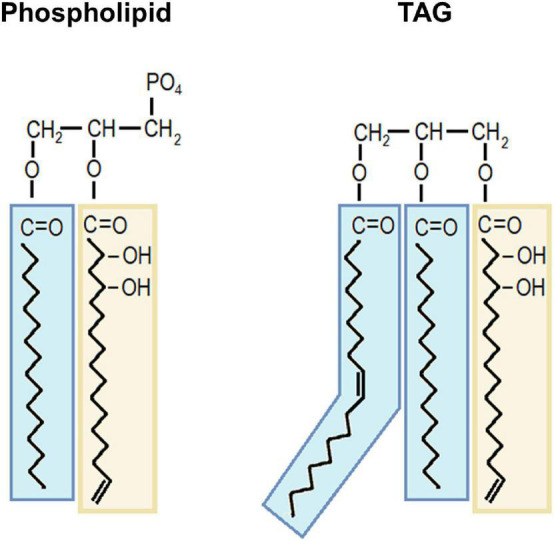
Proposed models of acetogenin moiety within polar lipids and triacylglycerols. A deacetylated acetogenin is depicted bound to a phospholipid as an example of polar lipid and to a TAG molecule. Avocadene backbone is shown as an acetogenin moiety example in yellow squares. Fatty acids are shown in blue squares. TAG, triacylglycerol.

Acetogenins found within lipids were identified in this work by the mass spectra of their odd acyl chain backbone (Supplementary Figure 2); thus, we cannot know if the whole AcO- group was attached in the complete molecule. This observation would imply that acetogenins form part of glycerolipids as an odd-acyl chain (probably hydroxylated), or that this and other editions (e.g., unsaturation, elongation) are happening while attached to the glycerol backbone. We consider this possibility, however, AcO-acetogenins contain the AcO- group attached to them α-carbon, making the esterification difficult in their acetylated form. In fact, other glycerolipids with acetoxy-FA acyl groups have been previously reported; however, those contain the AcO group in the β-carbon (Ayanoglu et al., 1985). Other examples are found in the oil of *Diascia* spp. which contains MAGs and DAGs with 3-acetoxy FAs (Dumri et al., 2008); TAGs from *Cardamine impatiens* seeds contain vicinal acetoxy FAs in the 13,14 positions (Mikolajczak et al., 1968). Based on these examples, we consider that esterification in the methyl end of the acetoxy group would be challenging; thus, acetogenins might be deacetylated for being bound to a glycerolipid (Figure 8). Very recently, the biological activity of deacetylated forms of acetogenins was reported (Tcheng et al., 2021). These forms inhibited FA oxidation, the mechanism by which cancer cell growth was decreased *in vitro*. On the other hand, their acetylated forms exhibited antimicrobials and antithrombotic activities (Rodríguez-Sánchez et al., 2013, 2015). Thus, the presence of these aliphatic molecules within lipids in avocado could act also as a way of regulating its role within avocado cells.

Regarding the relationship of free acetogenins and FAs in all tissues measured, the high number of paired quantitative analyses (112 total repetitions) allowed us to conduct multivariate analyses and visualize correlations among free FAs and AcO-acetogenins in avocado. Correlations were also performed for individual tissue samples; however, we selected the most conserved across avocado tissues by pooling all the data. It was not surprising to find a high number of significant correlations, as the finite FAs and lipid pools are metabolically quite close, consequently, saturated FAs clustered together. Nonetheless, the metabolic network that arose from this study showed interesting relationships. Firstly, linoleic acid, the only FA experimentally linked to AcO-persin (23:2) biosynthesis (Wang et al., 2004), appeared highly negatively correlated with this AcO-acetogenin (*r* = –0.75). Another FA with several connections was the omega-3 docosahexaenoic acid (DHA, 22:6) which negatively correlated with AcO-persenone A (23:3) and AcO-avocadene (19:1) (*r* = –0.73 and –0.70, respectively), the latter also negatively correlated with α-linolenic acid. Interestingly, the three closer FAs to AcO-acetogenins were unsaturated; this unsaturation was kept or diminished if we look at the first negatively correlated AcO-acetogenin. This observation prompts us to propose that immediate AcO-acetogenins precursors are unsaturated FAs, in which their unsaturation might stay in the acetoxy lipid.

On the other hand, it seems that AcO-persin is a common intermediary for shorter AcO-acetogenins. AcO-persin, thus, could be a substrate of acetogenin-editing enzymes, which modify it toward shorter and probably more available lipid forms for β-oxidation. The utilization of unusual FA’s as substrates for β-oxidation (specifically ricinoleic and vernolic acid) has been previously demonstrated in transgenic lines of *Arabidopsis* (Moire et al., 2004). AcO-acetogenins are not as abundant in grown leaves as they are in tissues with high metabolic demands (cotyledons and EA during germination and seedling development), in which AcO-persin was not as prevalent as with developed leaves. In fact, the number of carbons in the AcO-acetogenin moieties decreased during EA development. All these observations support the notion of AcO-acetogenins as essential components of avocado metabolism with more functions that merely serve as phytoalexins.

### Germination and Embryo Development in Avocado Involve a Continuous Carbon Allocation Within Lipids

Contrary to other seeds, mature avocado seeds are composed only by the embryo (cotyledons and EA) and a very thin dried seed coat as the remaining tissue of the integument and endosperm, which disappear in the mature seed (Piper and Gardner, 1943). Here, we are describing the changes in lipids between the two embryo tissues: cotyledons and EA. During seed imbibition (germination and post-germination), the avocado seed is composed of approximately 27–33% starch, 2% fat, 2.2–3.5% sugars, and 1.9–2.5% total proteins (Weatherby and Sorber, 1931; Bora et al., 2001). At the beginning of the avocado seed imbibition, sugars are immediately consumed in the cotyledon while starch contents steadily decline during the following days (Tesfay et al., 2012). We observed that total FAs remained unaltered in cotyledons during the 3 weeks of imbibition, while EA showed a significant decrease at 7–14 dai. Overall understanding of seed germination suggests that such reduction is likely a consequence of high TAG and FA breakdown to provide Acetyl-CoA for: (1) FA conversion to sucrose in the glyoxysomes, an essential pathway in early- and post-germination in *Arabidopsis thaliana* seeds (Eastmond, 2006), and (2) feeding the Krebs Cycle, which provides carbon skeletons and energy for the synthesis of fundamental structures like cell membranes and walls. Accordingly, embryo emergence, a process involving increased cell division and elongation, was observed between 7 and 14 dai, (see Supplementary Figure 3). Furthermore, lipid catabolism in seeds results in four-carbon intermediaries, which feed gluconeogenesis or support the respiratory chain (Theodoulou and Eastmond, 2012).

Interestingly, in cotyledons, both acetogenins and FAs increased after root protrusion (post-germination, from day 21 to 28), suggesting *de novo* lipid synthesis. The amount of starch accumulated in cotyledons might provide the carbon and energy to sustain this synthesis; moreover, the total carbon units allocated to both lipidic molecules in cotyledons during the 28 days of imbibition strongly supports this suggestion. FAs mobilization toward EA is hinted by the increase in carbon units toward FAs; also, a 1.4-fold rise in FAs in EA from 18 to 21 dai was observed. During post-germination, TAGs were reduced, and the PL pool was increased in cotyledons, while EA maintained the same pool of lipids, prevailing PLs, suggesting membrane synthesis. Similar tendencies were reported in seaberry (*Sea buckthorn)*, which fruit contains a considerable portion of oil in both mesocarp and seed and mobilizes TAGs and other glycerolipids from cotyledons during post-germination (Pchelkin et al., 2009).

In addition, our results showed a continuous edition of FAs and acetogenins, which are dynamically produced, elongated, and unsaturated during germination and development. Different forms of lipid edition, such as the interchange of acyl chains and desaturation, have been evidenced in germinating bean cotyledons and cucumber seeds (Murphy and Stumpf, 1980; Stymne and Glad, 1981). An increase in the FA pool of EA and cotyledon was seen in 28 dai in this study. Similar findings were observed in canola, alfalfa, and soybean seeds, where total FA content increased once the second germination phase concluded (Beevers, 1961; Ferrarese et al., 1998; Zhou et al., 2019).

Different reports on avocado seed FA profiles have determined oleic, linoleic, and palmitic as the most abundant FAs in avocado seed oil, in accordance with our results (Kilaru et al., 2015; Aliakbarzadeh et al., 2016; Ge et al., 2018). Besides avocado, other dicots like soy, canola, and *Arabidopsis* preferably accumulate linoleic acid during germination. Also, linoleic acid is the most abundant FA in phospholipids during germination of the said species (Joshi et al., 1973; Baleroni et al., 2000; Quettier and Eastmond, 2009). Our results showed that after 21 dai, imbibed cotyledons loose TAG contents. Interestingly, this is the time when the seeds showed a root protrusion in our samples. Storage tissues, such as cotyledons, generally start oil mobilization after root protrusion in oilseeds (Rosental et al., 2014), sea buckthorn (Pchelkin et al., 2009), and soybean (Yoshida, 1984). Such changes in oil profiles have also been reported in the starchy *Oryza* seeds as protrusion occurs (Kim et al., 2012).

This study provides the first evidence of the presence of acetogenins in developing EA. An interesting contrast was seen in the parallel determination of acetogenins and FAs in the EA. While the FA level rose at 28 dai, acetogenin level decreased, suggesting that the FAs released from triacylglycerols were used to provide energy for the seedling development and not acetogenin synthesis. Conversely, acetogenin content in cotyledon did not show reduction during the last weeks of imbibition. Although mobilization of reserves from cotyledon to EA is expected (Quettier and Eastmond, 2009), because of its large size, cotyledon harbors a much larger pool of triacylglycerols to be exploited, and thus, the reduction in acetogenin content was not observed.

As acetogenins are FA derivatives, precursors’ availability is determinant to their synthesis. Unsaturated FAs (e.g., linoleic and α-linolenic acids) are less available for breakdown and catabolism than saturated FAs, as their double bonds need to be isomerized from *cis*- to *trans*-composition (Engeland and Kindl, 1991). Being less available for breakdown, cells could use these unsaturated FAs to feed the pathway of acetogenin synthesis, which are always found unsaturated. In fact, palmitic and stearic acids decreased more than 34% each during the first week of imbibition in EA, while linoleic only decreased 23%. During germination, cotyledons continuously increased the carbon flux toward FAs and acetogenin synthesis. The increment of unsaturated FAs and acetogenins implies a continuous acyl edition during the germination process, probably to produce better substrates for energy production.

### Acetogenins Constitute a Significant Component of the Lipid Pool in Leaves

The acetogenin pool steadily increased during development in both seedling and tree leaves to contain a similar number of carbons allocated within them than within the FAs pool. Contrary to the lipids profiled in germinating avocado embryos, lipids profiled in leaves showed their accumulation in a photosynthetic tissue that will produce them with the carbon and energy obtained from CO_2_ fixation. True leaves settlement significantly impacts FA production, as part of it occurs in chloroplasts (Bao et al., 2000). Our findings through leaf development in seedlings evidence an enduring process by which the leaves continuously increase their acetogenin pool.

Avocado seedling leaves accumulated fewer FAs than tree leaves with a simpler set, showing a predominance of saturated FAs; only C18 FAs were unsaturated. Similar observations have been made in *Arabidopsis* leaves (Yang and Ohlrogge, 2009), *Malus x* Snowdrift *Cirsium vulgare*, and *Lactuca serriola leaves* (Lin and Oliver, 2008). The FA profile of tree leaves was more complex than that of seedling leaves in terms of length and unsaturation profiles. Temperature stress has been implicated in the unsaturation degree of the FAs of *A. thaliana* leaves. Heat treatment (37°C for two h) significantly increased unsaturated acyl chains found in TAGs compared to control plants (Mueller et al., 2015). Trees in this study were kept outside an average temperature of 32°C, and seed germination was performed in a growth chamber set at 25^°^C. Therefore, the temperature, plus the inherent developmental and growing differences, might be a factor in the desaturation degree from avocado leaves.

It was striking to observe that in both types of leaves, the carbon content allocated to form acetogenins was similar to the one comprising the FAs entire pool. Acetogenins found in lipid fractions did not follow the same accumulation pattern as free acetylated acetogenins; 82% of the pool in FGL consisted of avocadene and avocadyne, while AcO-avocadene and AcO-persenone A were the main acetylated acetogenins in FGL. Desaturases and other acyl editing enzymes prefer acyl chain substrates bound to PLs or galactolipids (Kazaz et al., 2021). As growing leaves do not need to store acyl chains in TAGs, PLs could be harboring acyl chains to be edited before being incorporated into TAGs. This mechanism is seen in the synthesis of ricinoleic acid, an unusual lipid, which is produced within PLs before being incorporated into TAGs (Kim et al., 2011).

Another particular feature of avocado leaf lipids was the prevalence of AcO-persin, which incremented during both seedling and tree leaves as they grew, making up to 69% of the pool in FGL. Although AcO-persin has been reported before in both avocado leaves (Carman and Handley, 1999) and fruits (Rodríguez-López et al., 2017), the latter contains less (4.5 vs. 1 mg/g). Thus, it is understandable that consumption of avocado leaves has been claimed to be toxic to some mammals while avocado mesocarp has not, as persin has been reported to induce necrosis in livestock (Butt et al., 2006).

According to our results for tree leaves, AcO-persin content also decreases as the leaves grow in the Fuerte avocado variety (Wang et al., 2004). Interestingly, young leaves from avocado seedlings followed an opposite tendency. The increment in acetogenins observed in seedling leaves was paralleled by a significant reduction of the major FAs (down to half from their initial content). Specifically, linoleic and α-linolenic acids suffered considerably decreases (4 to 2 and 5 to 1 mg/g DW, respectively) in the transition to full-grown leaves. This finding concurs with previous work where the substrate-product relationship between linoleic acid and AcO-persin was determined (Wang et al., 2004), suggesting that the carbon chain from linoleic acid is edited probably by hydroxylases to produce acetogenin molecules. The fact that this study blindly found this relationship in our correlation network plus other significant correlations among FAs and acetogenins strongly supports the other metabolic relations proposed here.

## Conclusion

Plants are able to synthesize a vast number of unusual lipids; to date, there is little knowledge about the role of these lipids in plant physiology.

Avocado, an ancient angiosperm, accumulates AcO-acetogenins throughout all tissues analyzed and during development. Remarkably, in leaves, we calculated that the amount of carbon within this odd-chain lipid was equivalent to that one allocated in FAs. We have also produced the first evidence of acetogenin backbones within lipids. Following acetogenin and FAs dynamics during germination and leaf development in seedlings and trees, we have uncovered possible metabolic connections, providing a picture of carbon allocation in lipids throughout these processes. All these together suggest that acetogenins could be used for providing energy and carbon units in plant metabolism, and maybe they have a role in signaling as they were widely found in PLs. Future experiments are needed to test both hypotheses.

This work also profiles for the first time FAs with such detail in avocado tissues other than fruit mesocarp. EA accumulated a myriad of FAs in small amounts during germination, which highly contrasts with the simple profile in leaves from seedlings. Fully photosynthetic leaves from trees accumulated more diverse FAs than seedlings, while cotyledons kept synthetizing FAs and acetogenins after root protrusion. The information provided by this work sets a foundation for studying further unusual lipids in avocado and maybe other plants. AcO-acetogenins are enigmatic molecules with several potential uses in the pharma, cosmetics, and food industries. Thus, knowledge about their *in planta* metabolism will contribute to their development as ingredients in a sustainable form.

## Data Availability Statement

The original contributions presented in the study are included in the article/Supplementary Material, further inquiries can be directed to the corresponding author/s.

## Author Contributions

ÁC-O and RD contributed to the conception and design of the study. ÁC-O, SG-A, LM-O, and DR-S performed the experiments. ÁC-O and VT performed the statistical analysis. ÁC-O, SG-A, and RD wrote the first draft of the manuscript. CH-B, DR-S, and VT wrote sections of the manuscript. CH-B, DR-S, LM-O, and RD designed the methodologies for lipid fractionation and acetogenin identification. All authors contributed to the discussion of data, manuscript revision, read, and approved the submitted version.

## Conflict of Interest

CH-B, DR-S, and RD are inventors for several acetogenins in the food, pharmaceutical, and personal care industries, which are under patent applications PCT/IB2011/053535 and PCT/IB2015/002021 and US2018013671A1 and MX 3355202 B. The remaining authors declare that the research was conducted in the absence of any commercial or financial relationships that could be construed as a potential conflict of interest.

## Publisher’s Note

All claims expressed in this article are solely those of the authors and do not necessarily represent those of their affiliated organizations, or those of the publisher, the editors and the reviewers. Any product that may be evaluated in this article, or claim that may be made by its manufacturer, is not guaranteed or endorsed by the publisher.

## Footnotes

1
https://cran.r-project.org/

## Supplementary Material

Supplementary Figure 1 Examples of plant tissues used for acetogenin and fatty acid analyses.

Supplementary Figure 2 Potential sites of O-methylation of AcO-Avocadene methyl ether.

Supplementary Figure 3 Avocado seed imbibition.

Supplementary Figure 4 Correlation networks from avocado individual tissue samples.

Supplementary Table 1 Acetogenin structures.

Supplementary Table 2 Percent distribution of an acetogenin-enriched extract (Avosafe^®^) separated in five lipid subfractions by solid phase extraction.

Supplementary File 1 Acetogenin content and composition in different avocado tissues (mg g^−1^ DW).
