# Supplementary material for: The Enigmatic Aliphatic Acetogenins and Their Correlations With Lipids During Seed Germination and Leaf Development of Avocado (Persea americana Mill.)
Source: Front Plant Sci. 2022 May 3;13:839326. doi: 10.3389/fpls.2022.839326 (PMC9111537; doi:10.3389/fpls.2022.839326)
Supplement: Supplementary Figure 1 — Examples of plant tissues used for acetogenin and fatty acid analyses. [file Data_Sheet_1.pdf]

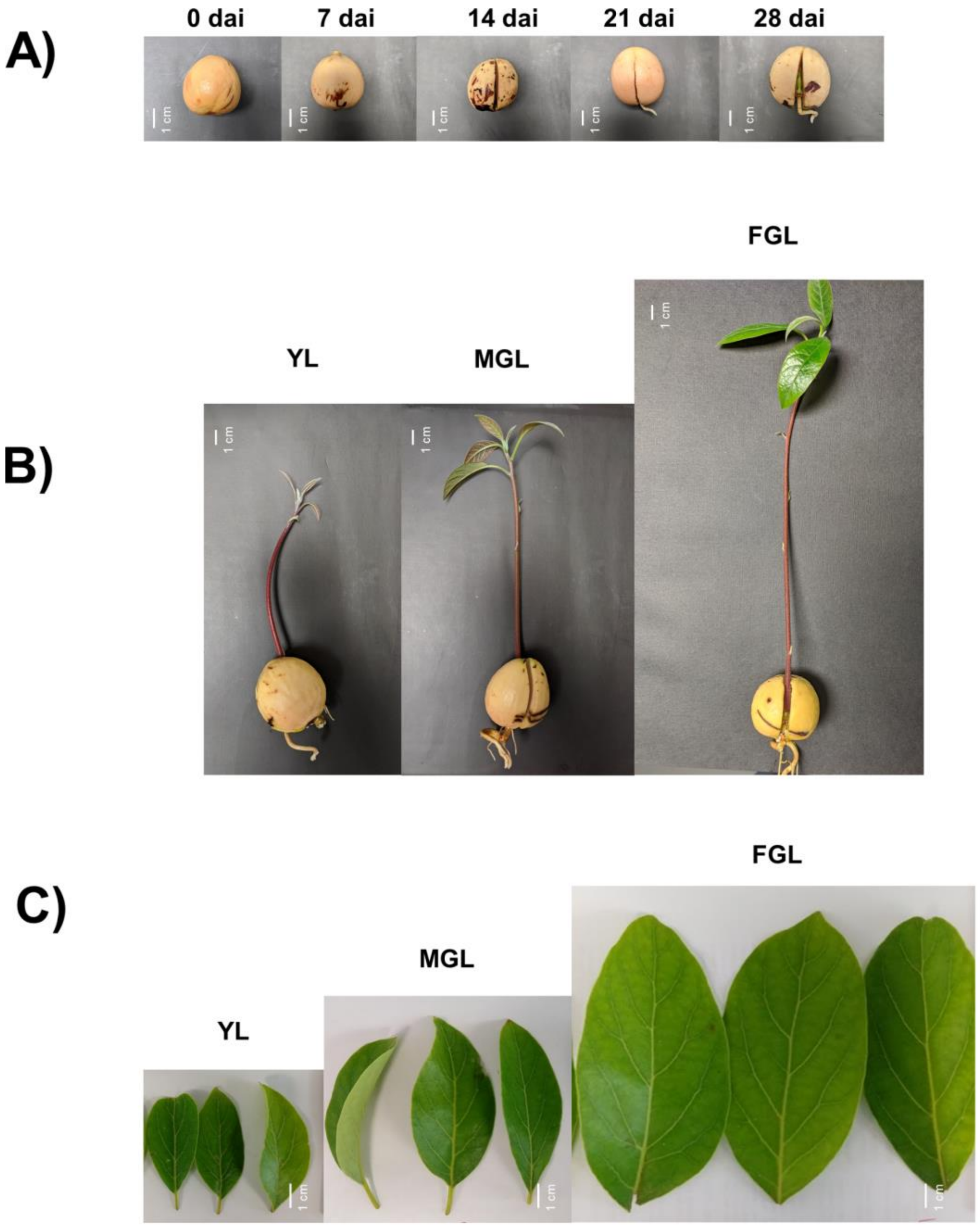

Supplementary Figure S1. Examples of plant tissues used for acetogenin and fatty acid analyses. A) Avocado seeds maintained in imbibition. B) Avocado seedlings used for the collection of seedling leaves. C) Leaves collected from avocado trees. dai, days after imbibition; YL, young leaf; MGL, middle-grown leaf; FGL, full-grown leaf

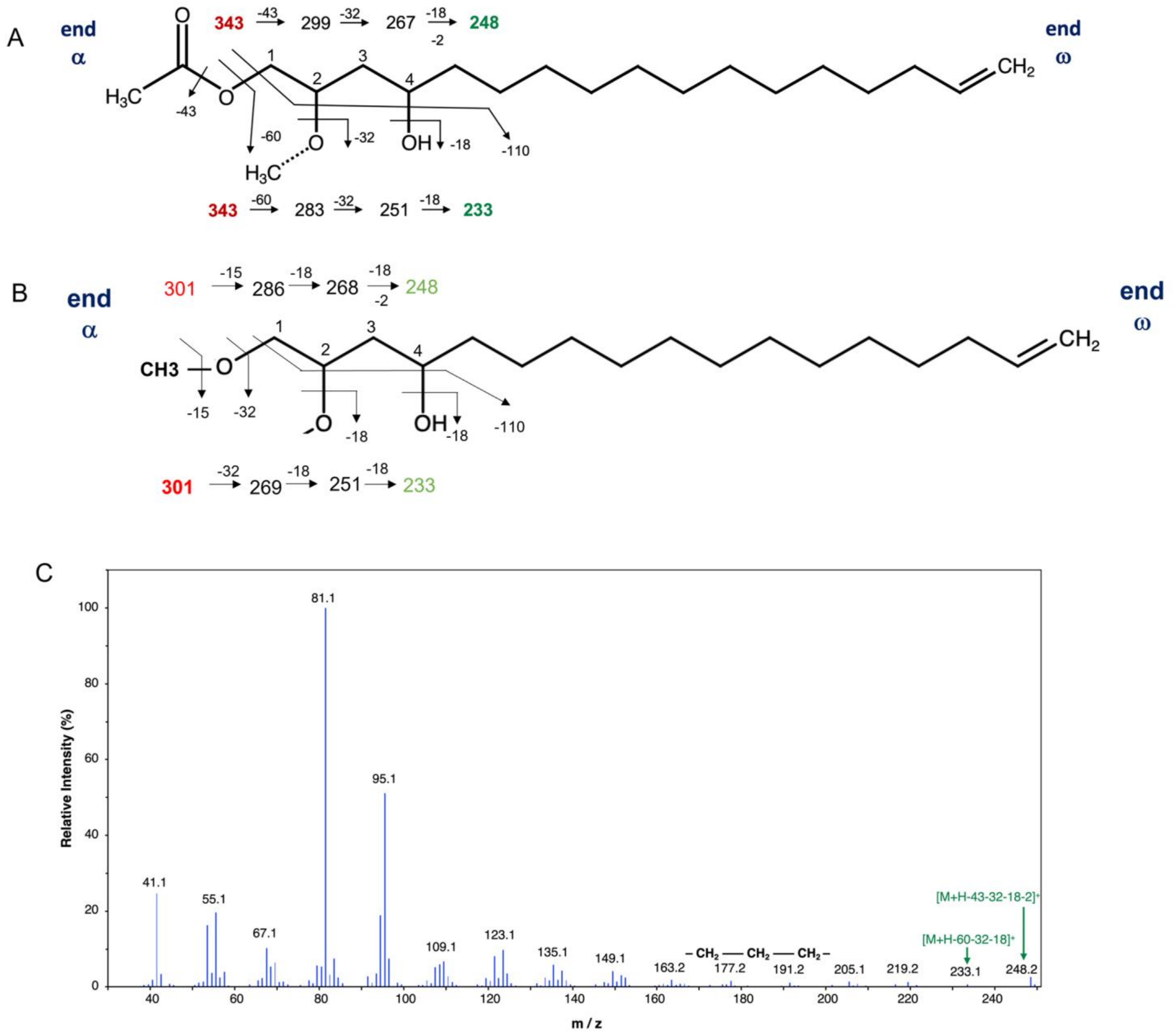

Supplementary Figure S2. Potential sites of O-methylation of AcO-avocadene methyl ether. At C-2 (A) or at C-1 (B) and GC-MS spectra observed by electron impact (EI) ionization mode, 70 eV (C)

Figure S2A and B shows the potential sites of O-methylation for AcO-avocadene. The presence of a strong acid (sulfuric acid) and methanol during derivatization, mediates activation of hydroxyls groups (OH) groups of acetogenins (in C2 and C4 or in a β-hydroxy group carbonyl system) and methanol (Busta, Budke, & Jetter, 2016; Iqbal & Engineering, 2014; Wu & Sun, 2005). This converts such OHs into good leaving groups (Ciucanu, 2006), easing O-methylation in OH at C-2 (Figure S2A), and therefore allowing acetogenin detection by GC as methyl ethers (Siano et al., 2003).

Moreover, it has been reported that derivatization of β-hydroxy carboxylic acids, preferentially takes place at the carboxylic hydroxyl group (OH), retaining OH attached to the β-carbon (Wu & Sun, 2005). In the case of acetogenins, the resembling carboxylic OH is protected by an acetyl (acetoxy group) and bounded to the aliphatic chain, reducing its reactivity (Tsukamoto, Itoh, Kobayashi, & Obora, 2019). However, OH at C-1 can be another possible site of O-methylation (Figure S2B), similarly to the reported for β-hydroxy carboxylic acids, ester-linked to glucosamines of lipid A, which also preserve the OH attached to the β-carbon (Iqbal & Engineering, 2014).

The mass spectra of AcO-avocade methyl ether, together with the spectra of other seven free AcOMEs derived from Avosafe® showed a characteristic mass fragmentation pattern (Figure S2C). Firstly, the strong ionization energy of EI and the lability of oxygenated groups (acetoxy, hydroxyls, carbonyl) associated to Mclafferty rearrangement characteristic of esters in the acetoxy group (Mclafferty & Gohlke, 1959; Weiler, 1972), made difficult to observe the parent ions corresponding to the methyl ether.

Particularly, as shown in Figure S2A, a diagnostic fragment consistently present in the mass spectra of different AMEs (O-methylation occurring in OH at C-2) corresponded to a loss of *m/z* 110 from the methylated parent molecule (M+H-110). potentially due to the progressive losses of hydroxy (-OH, *m/z* 18), acetoxy ($-COOHCH_3$, *m/z* 60) and methoxy groups ($-OCH_3$, *m/z* 32). The loss of an extra hydrogen appreciable for the acetoxyl, hydroxyl and methoxyl can be attributed to the ortho-effect (Mclafferty & Gohlke, 1959). In the case of O-methylation occurring in OH at C-1, this loss corresponded to a fragment *m/z* 68, resulting from losses of methoxyl ($-OCH_3$, *m/z* 32) and two hydroxyls (-OH, *m/z* 18, each), as shown in Figure S2B. The loss of *m/z* 110 and *m/z* 68 from methylated AcO-Avocadene or methylated Avocadene, respectively, produced a common peak at *m/z* 233 (Figure S2C). Interestingly, FAMEs also present losses of methoxy group ($-OCH_3$, *m/z* 32), however presence of a carboxyl head group, instead of an acetoxyl characteristic acetogenins, produces Mclafferty rearrangement ions with m/z 74 and m/z 87, or stable M+H-74 o M+H-87 (Iqbal & Engineering, 2014; Murad et al., 2014), not visible for acetogenin. Other characteristic ion was M+H-95, when O-methylation occurred in OH at C-2, likely arising from progressive losses of acetyl ($-COCH_3$, *m/z* 43), methoxyl ($-OCH_3$, *m/z* 32), hydroxyl (-OH, *m/z* 18) and loss of 2H, attributed to rearrangement of the remaining ion as a ketene ($[M-H-R-CH=C=O]^+$) (Hsu & Turk, 2001). When O-methylation occurred in OH at C-1, this fragment ion was M+H-53, matching losses of a methyl ($-CH_3$, *m/z* 15), two hydroxyl (-OH, *m/z* 18, each) and loss of 2H for a ketene rearrangement. These losses of *m/z* 95 and *m/z* 53 from methylated AcO-Avocadene or methylated Avocadene, respectively, produced a common peak at *m/z* 248, as well as peak at *m/z* 95 (Figure S2C).Additionally, a series of ions was also detected based on the M+H-110 m/z ion or M+H-95, showing repetitive differences of m/z 14 (Figure S2C), characteristics of consecutive losses of methylene group ($-CH_2-$) of the hydrocarbon chain (Iqbal & Engineering, 2014), interrupted by the presence of C-C unsaturations.

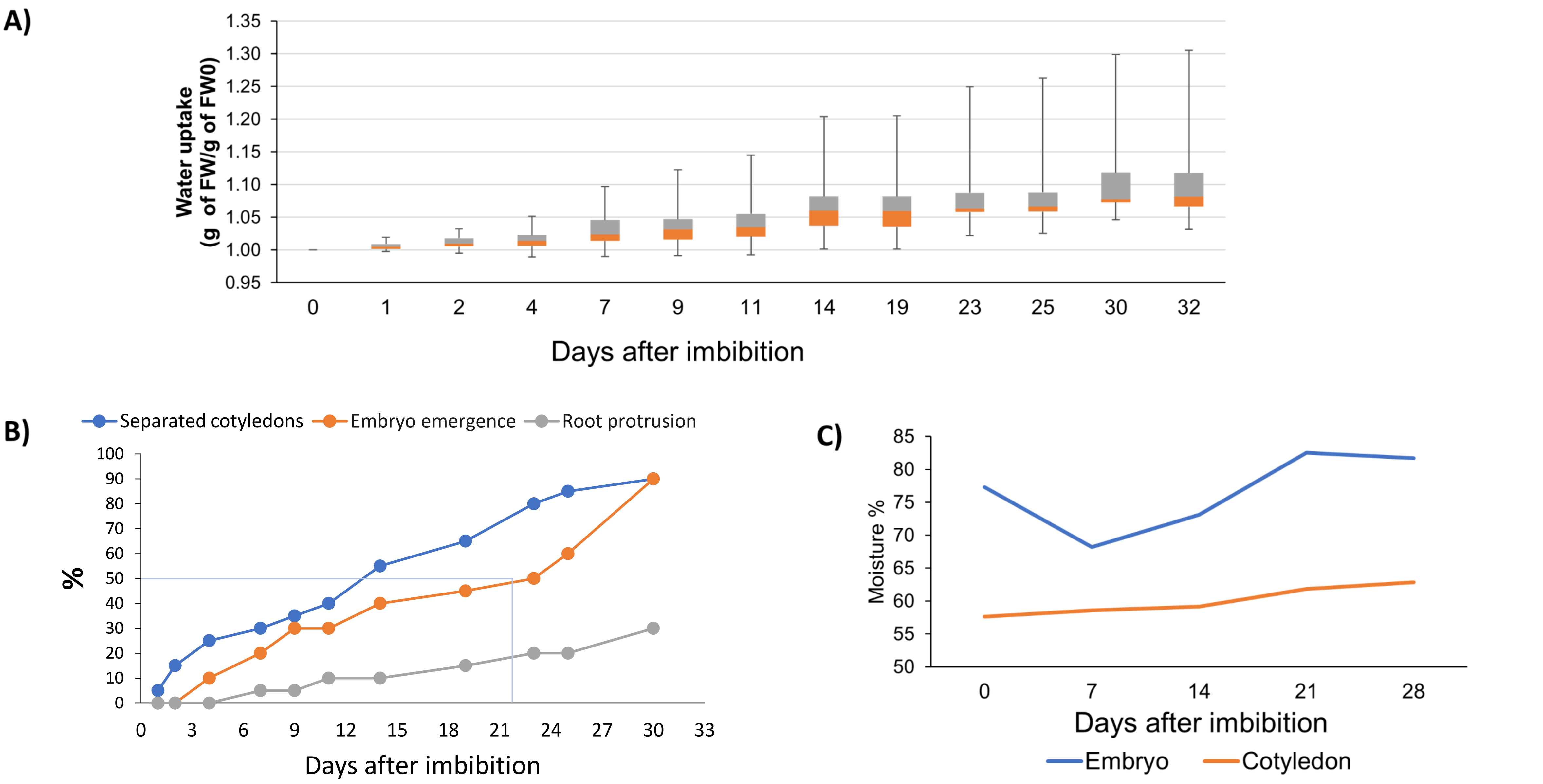

Supplementary Figure S3. Avocado seed imbibition. A) Water uptake at different days after imbibition (DAI). B) Seed percentage with separated cotyledons, embryo emergence, and root protrusion at different DAI. C) Moisture of embryo axe and cotyledons at different DAI. N = 20, Seed FW, fresh weight (g); FW0, fresh weight (g) at day 0.

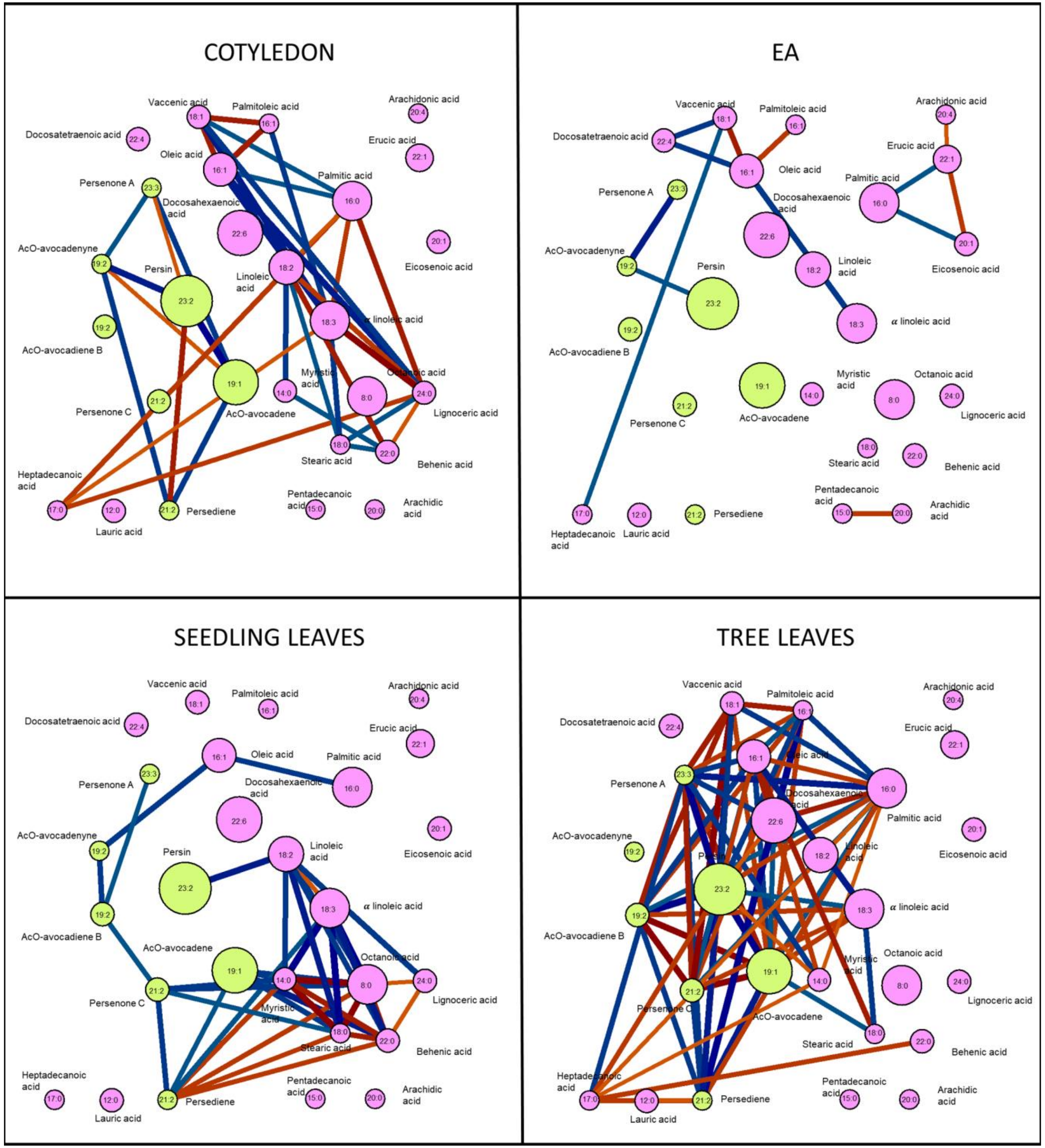

Supplementary Figure S4. Correlation networks from avocado individual tissue samples. Each network was formed after removing absolute correlations lower than 0.66. Edges color (red, positive; blue, negative) and width represent the correlation. Nodes represent metabolites as labeled; size is proportional to connections. Pink for fatty acids and green for acetogenins. Data were obtained from 19 samples and 38 determinations for cotyledons and EA (each), and 9 samples and 18 determination for each set of leaves.
