# Supplementary material for: The Enigmatic Aliphatic Acetogenins and Their Correlations With Lipids During Seed Germination and Leaf Development of Avocado (Persea americana Mill.)
Source: Front Plant Sci. 2022 May 3;13:839326. doi: 10.3389/fpls.2022.839326 (PMC9111537; doi:10.3389/fpls.2022.839326)
Supplement: Supplementary Table 1 — Acetogenin structures. [file Data_Sheet_2.pdf]

Supplementary Table S1. Acetogenin Structures

Compound	Carbon: Unsaturation	Structure
**Avocadyne**	**19:1**	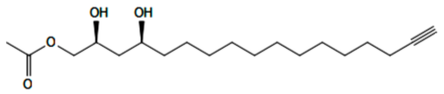
**Avocadene**	**19:1**	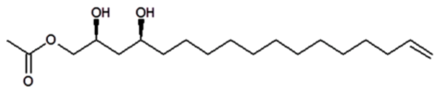
**Avocadenyne**	**19:2**	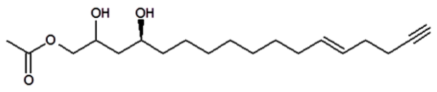
**Avocadiene-B**	**19:2**	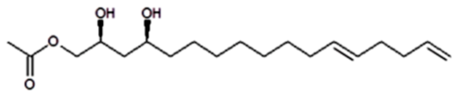
**Persenone B**	**21:1**	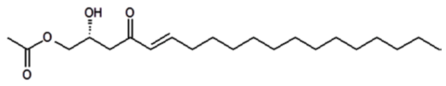
**Persenone C**	**21:2**	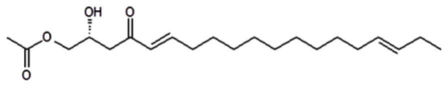
**Persediene**	**21:2**	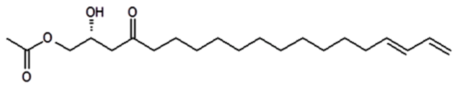
**Persin**	**23:2**	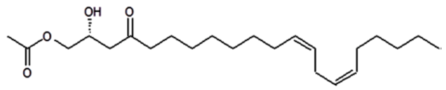
**Persenone A**	**23:3**	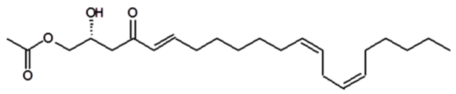

Supplementary Table S2 Percent distribution of an acetogenin-enriched extract (Avosafe®) separated in five lipid subfractions by solid phase extraction.

External Standard	Distribution %[1]				
	F1-S	F2-TG	F3-MG+DG	F4-FFA	F5-PL
Avosafe®	19.0	0.5	68.2	2.0	1.7

Avosafe®= Food-Grade Acetogenin-Enriched Extract from avocado seed. ACE=acetogenins; F= fraction; S= Sterols; TG=Triglycerides; MG= monoglycerides; DG= Diglycerides; FFA=Free fatty acids; PL=phospholipids.

[1]Percent acetogenin concentration in each subfractions was calculated based on the total acetogenin concentration of the non-fractionated external standard.
